# Aberrant Brain Regional Homogeneity and Functional Connectivity in Middle-Aged T2DM Patients: A Resting-State Functional MRI Study

**DOI:** 10.3389/fnhum.2016.00490

**Published:** 2016-09-27

**Authors:** Daihong Liu, Shanshan Duan, Jiuquan Zhang, Chaoyang Zhou, Minglong Liang, Xuntao Yin, Ping Wei, Jian Wang

**Affiliations:** ^1^Department of Radiology, Southwest Hospital, Third Military Medical UniversityChongqing, China; ^2^Department of Endocrinology, Southwest Hospital, Third Military Medical UniversityChongqing, China

**Keywords:** type 2 diabetes mellitus, resting-state functional magnetic resonance imaging, regional homogeneity, functional connectivity, cognitive impairment

## Abstract

Type 2 diabetes mellitus (T2DM) has been associated with cognitive impairment. However, its neurological mechanism remains elusive. Combining regional homogeneity (ReHo) and functional connectivity (FC) analyses, the present study aimed to investigate brain functional alterations in middle-aged T2DM patients, which could provide complementary information for the neural substrates underlying T2DM-associated brain dysfunction. Twenty-five T2DM patients and 25 healthy controls were involved in neuropsychological testing and structural and resting-state functional magnetic resonance imaging (rs-fMRI) data acquisition. ReHo analysis was conducted to determine the peak coordinates of brain regions with abnormal local brain activity synchronization. Then, the identified brain regions were considered as seeds, and FC between these brain regions and global voxels was computed. Finally, the potential correlations between the imaging indices and neuropsychological data were also explored. Compared with healthy controls, T2DM patients exhibited higher ReHo values in the anterior cingulate gyrus (ACG) and lower ReHo in the right fusiform gyrus (FFG), right precentral gyrus (PreCG) and right medial orbit of the superior frontal gyrus (SFG). Considering these areas as seed regions, T2DM patients displayed aberrant FC, mainly in the frontal and parietal lobes. The pattern of FC alterations in T2DM patients was characterized by decreased connectivity and positive to negative or negative to positive converted connectivity. Digital Span Test (DST) forward scores revealed significant correlations with the ReHo values of the right PreCG (ρ = 0.527, *p* = 0.014) and FC between the right FFG and middle temporal gyrus (MTG; ρ = −0.437, *p* = 0.048). Our findings suggest that T2DM patients suffer from cognitive dysfunction related to spatially local and remote brain activity synchronization impairment. The patterns of ReHo and FC alterations shed light on the mechanisms underlying T2DM-associated brain dysfunction and might serve as imaging biomarkers for diagnosis and evaluation.

## Introduction

Type 2 diabetes mellitus (T2DM) is characterized by progressive insulin secretion defects on the basis of insulin resistance (American Diabetes Association, 2015) and is accompanied by hyperinsulinemia, hyperglycemia, increased inflammatory mediators, advanced glycation end products and amyloid deposition. These risk factors have caused T2DM to become closely associated with cognitive dysfunction, which can even progress to dementia (Strachan et al., 2011). Data from the International Diabetes Federation Annual Report^1^ showed that as many as 415 million people were living with diabetes in 2015. With a risk of dementia greater than 6–7% (Koekkoek et al., 2015), the report called for effective means to cope with the emerging novel complications. It is highly important to illuminate the neurological mechanisms of cognitive dysfunction induced by T2DM, as this could enable the application of preventive treatment strategies.

Resting-state functional magnetic resonance imaging (rs-fMRI) can provide excellent opportunities to investigate cognitive dysfunction related to T2DM *in vivo* (Musen et al., 2012; Xia et al., 2013; Chen et al., 2014; Cui et al., 2014, 2015). Among several rs-fMRI indices, regional homogeneity (ReHo) and functional connectivity (FC) are often used to evaluate brain activity synchronization in normal subjects or patients. ReHo measures the similarity of the time series of a given voxel to its nearest neighborly voxels, and it is related to local synchronization (Zang et al., 2004). There were only two studies which investigated ReHo changes in T2DM patients (Cui et al., 2014; Peng et al., 2016). Cui et al. (2014) reported a decrease of ReHo primarily in the occipital lobe that was related to poor cognitive performance in T2DM patients. Peng et al. (2016) revealed abnormal brain activity in T2DM patients with and without microangiopathy using ReHo analysis. It appears that the occipital lobe, temporal lobe, precuneus, frontal gyrus and insula brain regions are susceptible to T2DM (Peng et al., 2016). Regarding FC, it measures the similarity of the time series of two relatively remote brain regions (Biswal et al., 1995). Alterations in the FC of the default mode network (DMN) were recently investigated using independent component analysis (Cui et al., 2015). T2DM patients exhibited increased connectivity in the anterior sub-network of the DMN, but decreased connectivity in the posterior sub-network of the DMN (Cui et al., 2015).

As effective indicators reflecting the intrinsic organization of the resting brain, ReHo and FC have been conjunctively applied in a number of studies of complex brain functional activity and its alterations in diseases and aging (Damoiseaux et al., 2008; Jiang et al., 2015; Cui et al., 2016; Wang et al., 2016). These two indices have proved to be mutually complementary for detecting local and remote brain activity synchronization. However, the aberrant brain function activity in middle-aged T2DM patients has not been elucidated using these two methods together. Therefore, it has been suggested that the combination of ReHo and FC analyses could provide more information about aberrant brain activity synchronization in T2DM patients than either index alone.

Based on the above-mentioned findings, we hypothesized that T2DM relates to aberrant spatially local and remote brain activity synchronization that might be associated with cognitive impairment. In the present study, we first investigated the abnormal brain activity using ReHo analysis. According to the peak Montreal Neurological Institute (MNI) coordinates of brain regions with abnormal ReHo, we further explored the FC between these brain regions and all other brain voxels. Finally, we investigated the relationships of aberrant ReHo and FC with neuropsychological test scores and diabetes-related parameters. Our exploratory study provides complementary information for a deeper understanding of the mechanisms underlying T2DM-associated brain dysfunction.

## Materials and Methods

### Subjects

The subjects were recruited from inpatients and communities between December 2013 and January 2015. The T2DM patients met the criteria published by the World Health Organization in 1999 (Alberti and Zimmet, 1998), and they were diagnosed with diabetes for more than 2 years. All of the subjects were right handed, between the ages of 45 and 60 years old and had at least 6 years of education. Healthy controls and T2DM patients were group-matched by age, sex, education level and body mass index (BMI). The exclusion criteria for all subjects were a history of stroke, white matter hyperintensity showing confluence or severe changes (Wahlund et al., 2001; Chen et al., 2014), Parkinson’s disease, probable dementia (Mini-Mental State Examination [MMSE] score ≤24; Galea and Woodward, 2005), depression (Hamilton Depression Rating Scale [HAMD] >7; Hamilton, 1960) or any other neuropsychiatric disorder, thyroid dysfunction, severe renal impairment, severe visual or hearing loss and any contraindications to MRI. Of the 64 recruited subjects, 25 T2DM patients and 25 healthy controls were screened for subsequent studies (Table 1). All the participants provided written informed consent, and the study was approved by the local Medical Research Ethics Committee of the Southwest Hospital (Chongqing, China).

**Table 1:** **Demographic and clinical data comparison results of T2DM patients and healthy controls**.

	T2DM	HC	*p* value
Age (years)	52.24 ± 4.78	52.08 ± 3.46	0.893
Sex (male:female)	17/8	13/12	0.248^a^
Education level (years)	11.04 ± 3.05	11.16 ± 2.34	0.877
T2DM duration (years)	7.72 ± 5.44	−	−
BMI (kg/m^2^)	24.69 ± 3.25	25.05 ± 2.88	0.683
Systolic blood pressure (mm Hg)	124.72 ± 13.69	126.16 ± 12.60	0.701
Diastolic blood pressure (mm Hg)	79.36 ± 8.02	77.04 ± 7.10	0.284
HbA_1c_ (%)	8.40 (7.40, 9.60)	5.70 (5.40, 6.00)	<0.001*^b^
HbA_1c_ (mmol/mol)	68.00 (57.00, 81.50)	39.00 (35.50, 42.00)	<0.001*^b^
Fasting plasma glucose (mmol/L)	7.41 (5.28, 8.80)	5.28 (4.86, 5.50)	0.002*^b^
Fasting insulin (mIU/L)	12.00 (8.94, 17.64)	10.02 (7.78, 17.41)	0.352^b^
Fasting C-peptide (ng/ml)	1.85 ± 1.20	2.31 ± 1.14	0.175
Total cholesterol (mmol/L)	4.91 ± 1.28	5.20 ± 0.94	0.368
Triglyceride (mmol/L)	1.37 (0.93, 2.29)	1.37 (1.06, 1.85)	0.985^b^
HDL cholesterol (mmol/L)	1.10 ± 0.20	1.37 ± 0.34	0.002*
LDL cholesterol (mmol/L)	3.10 ± 0.10	3.32 ± 0.72	0.382
Homocysteine (μmol/L)	13.89 ± 8.13	12.12 ± 4.08	0.336
Blood urea nitrogen (mmol/L)	5.58 ± 1.74	5.43 ± 1.09	0.706
Serum creatine (μmol/L)	65.60 ± 15.86	75.92 ± 14.43	0.020*
Urinary microalbumin (mg/dL)	0.90 (0.60, 1.85)	1.20 (0.80, 1.65)	0.280^b^
Cystatin C (mg/L)	0.82 ± 0.44	0.71 ± 0.15	0.243
Uric acid (μmol/L)	304.16 ± 66.21	328.64 ± 61.95	0.183
FT3 (pmol/L)	4.10 ± 0.81	5.20 ± 0.58	<0.001*
FT4 (pmol/L)	15.18 ± 2.18	16.29 ± 1.91	0.061
TSH (mIU/L)	2.10 ± 0.78	2.02 ± 1.05	0.761
Gray matter (cm^3^)	613.10 ± 49.70	632.60 ± 44.00	0.147
White matter (cm^3^)	529.20 ± 57.10	540.30 ± 71.10	0.546
Brain parenchyma (cm^3^)	1142.30 ± 104.20	1172.90 ± 103.10	0.302

*p < 0.05. Data are presented as n for proportions, means ± SD for normally distributed continuous data and median (QR) for non-normally distributed data. ^a^The p value for sex was obtained using the χ^2^ test. ^b^The p value was obtained using the Mann-Whitney U test. T2DM, type 2 diabetes mellitus; BMI, body mass index; HDL, high density lipoprotein; LDL, low density lipoprotein; FT3, free triiodothyronine; FT4, free thyroxine; TSH, thyroid stimulating hormone.

### Clinical Data

Data on height, weight and arterial blood pressure were obtained by measurements on the spot. Medical history and medication use were acquired from medical records, questionnaires and interviews. The T2DM patients were undergoing treatment with one or more of the following medications: hypoglycemic agents (insulin, *n* = 12; biguanides, *n* = 14; sulfonylureas, *n* = 8; glinides, *n* = 2; α glucosidase inhibitors, *n* = 5; thiazolidinediones, *n* = 3); antihypertensives (angiotensin II receptor blockers, *n* = 3; angiotensin-converting enzyme inhibitors, *n* = 1; calcium channel blockers, *n* = 1); and statins (*n* = 2). Then, BMI was calculated ([weight in kg]/[height in m]^2^). Blood samples were collected by venipuncture for the measurement of fasting plasma glucose, fasting insulin, fasting C-peptide, glycosylated hemoglobin (HbA_1c_), triglycerides, total cholesterol, triglyceride, high density lipoprotein (HDL) cholesterol, low density lipoprotein (LDL) cholesterol, homocysteine, blood urea nitrogen, serum creatine, urinary microalbumin, cystatin C, uric acid, free triiodothyronine (FT3), free thyroxine (FT4) and thyroid stimulating hormone (TSH) levels, with the patient in the fasting state after overnight abrosia. Because of the influence of exogenous insulin (primarily the injection of insulin) on fasting plasma insulin levels, the score for homeostasis model assessment of insulin resistance was not calculated.

### Neuropsychological Tests

The cognitive status of the participants was evaluated by a battery of neuropsychological tests covering major cognitive domains. The MMSE and Montreal Cognitive Assessment (MoCA) tests were conducted to evaluate the general level of cognition. The HAMD was used to exclude possible cases of depression. Then, attention, executive function and psychomotor speed were evaluated by the Trail Making Test parts A and B (TMT-A and TMT-B; Bowie and Harvey, 2006). Working memory was evaluated by the Digital Span Test (DST, forwards and backwards; Gong, 1992). Semantic memory was evaluated by the Verbal Fluency Test (VFT; Brucki and Rocha, 2004). Episodic memory was evaluated by the Auditory Verbal Learning Test (AVLT, including immediate recall, short term delayed recall, long term delayed recall, long term delayed recognition and total score; Schmidt, 1996). All of the tests were administered by a trained neuropsychologist who was blinded to the subjects’ diagnoses. Each individual required approximately 60 min to complete the test in a fixed order.

### MRI Data Acquisition

MRI data were acquired using 3.0-T MR scanner (Trio, Siemens Medical, Erlangen, Germany) with a 12-channel head coil at approximately 9:00 a.m. after clinical data collection and neuropsychological tests. Earplugs were used to diminish the influence of scanner noise. All of the subjects were instructed to remain awake with their eyes closed and to not think about anything in particular. T2-weighted and fluid attenuated inversion recovery (FLAIR) images were obtained for the assessment of white matter hyperintensity and intracranial structural lesions. High-resolution T1-weighted images were obtained sagittally using a volumetric 3D magnetization prepared rapid gradient-echo sequence: TR 1900 ms, TE 2.52 ms, flip angle 9°, FOV 256 mm × 256 mm, slices = 176, thickness = 1 mm, in-plane matrix = 256 × 256 and voxel size = 1 mm × 1 mm × 1 mm. Resting-state functional images were obtained transversely using an echo planar imaging (EPI) sequence: TR 2000 ms, TE 30 ms, flip angle 90°, FOV 192 mm × 192 mm, slices = 36, thickness = 3 mm, in-plane matrix = 64 × 64 and voxel size = 3 mm × 3 mm × 3 mm; 240 volume acquisition cost 8 min and 8 s.

### Data Analysis

The T2-weighted and FLAIR images were separately reviewed by two experienced neurologists blinded to the group status. The two neurologists reached consensus on brain disease assessment through discussion. T1-weighted images were processed by the VBM 8 toolbox, based on Statistical Parametric Mapping 8 software (SPM 8, Wellcome Department of Imaging Neurosciences, University College London, UK^2^) running on MATLAB 7.14 (Math-Works, Natick, MA, USA^3^). The process obeyed the following standard protocol (Whitwell, 2009). Intracranial tissue was segmented into gray matter, white matter and cerebrospinal fluid. The volumes of the gray matter, white matter and cerebrospinal fluid were automatically calculated, and the brain parenchyma volume was equal to the sum of the gray matter and white matter volumes. Functional image preprocessing and ReHo calculation were conducted with Data Processing Assistant for Resting-State fMRI (DPARSF v2.3^4^). The first 10 volumes of functional images were removed for their possible heterogeneity. Slice-timing and realignment were performed for head motion using the remaining images. No subjects were excluded from the analyses due to head motion >2.0 mm in any direction of *x, y* and *z* or 2.0° of any angular motion. Then, the functional images were coregistered to the high-resolution T1-weighted images. Subsequently, the coregistered images were normalized to the MNI template. Detrending was performed to remove linear trends from the image time series, and the data were filtered at the 0.01–0.08 Hz band. ReHo calculation was performed using the preprocessed images, and the resulting images were smoothed with an isotropic Gaussian kernel of 4 mm full-width half-maximum.

FC calculation was conducted with rs-fMRI data analysis toolkits (REST v1.8^5^). The coordinates of the center of the seed region were based on the inter-group analysis of ReHo, and the radius was 4 mm. Correlation analysis of time course was performed between the spherical seed region and every voxel of the whole brain for each subject. Finally, Fisher’s *r*-to-*z* transformation was applied to improve the normality of the ReHo and FC maps (Song et al., 2011).

### Statistical Analysis

Demographic/clinical data and neuropsychological test scores were compared between the T2DM patients and healthy controls using SPSS software (version 20.0; SPSS Inc., Chicago, IL, USA). We used the χ^2^ test for proportions, the independent samples *t*-test for normally distributed continuous data and the Mann-Whitney U test for non-normally distributed data. The distribution of the data was analyzed using the Kolmogorov-Smirnov test. A *p*-value < 0.05 was considered statistically significant.

ReHo and FC map analyses were conducted with REST software. Jarque-Bera goodness-of-fit test was applied to test the distribution of ReHo and FC maps within 61 × 73 × 61 brain mask (70,831 voxels). The results showed that both ReHo and FC maps were normally distributed (100% voxel with *p* > 0.05). Intra-group patterns were analyzed using the one-sample *t*-test, with ReHo maps compared to “1” (Chao-Gan and Yu-Feng, 2010) and FC maps compared to “0”. The inter-group differences on the ReHo and FC maps were analyzed using the two-sample *t*-test. Age, sex, education and BMI were imported as covariates. Gray matter maps obtained from VBM analysis were also used as covariates to exclude the possible effects of gray matter atrophy on the results. The voxel level was *p* < 0.005. The outcome maps of ReHo and FC were then corrected with AlphaSim (cluster *p* < 0.01, cluster size >16 voxels, full width at half maximum = 4 mm, number of Monte Carlo simulations = 1000, cluster connection radius: rmm = 5.00) to detect the brain regions that survived the comparison and to determine their peak MNI coordinates. Finally, the *z* scores of abnormal brain regions were extracted for subsequent correlation analyses.

To explore the relationships among ReHo values, FC *z* scores, neuropsychological performances and diabetes-related parameters in T2DM patients, partial correlation analyses were conducted with SPSS software (age, sex, education and BMI were included as control variables).

## Results

### Demographic and Clinical Data

The subjects’ demographic and clinical characteristics are reported in Table 1. The two groups did not show significant differences in terms of age, sex, education level, BMI, systolic/diastolic blood pressure, total cholesterol, triglycerides, LDL cholesterol, homocysteine, blood urea nitrogen, urinary microalbumin, cystatin C, uric acid, FT4 or TSH (all *p* > 0.05). However, the T2DM patients exhibited significantly higher HbA_1c_ and fasting plasma glucose than healthy controls, but lower HDL cholesterol and serum creatine than healthy controls (all *p* < 0.05). The FT3 levels of the T2DM patients were significantly higher than those of the healthy controls (*p* < 0.05) but within the normal range. No subject was excluded for severe white matter hyperintensity or intracranial lesions.

### Neuropsychological Test Results

The T2DM patients scored poorer on the TMT-A, TMT-B and DST forward tests (all *p* < 0.05) and had no significant decreases in the other neuropsychological tests (all *p* > 0.05; Table 2).

**Table 2:** **Comparison of neuropsychological test results of T2DM patients and healthy controls**.

	T2DM	HC	*p* value
**General cognition**
MMSE	27.92 ± 1.91	28.20 ± 1.50	0.567	
MoCA	23.04 ± 2.85	24.44 ± 2.57	0.074
**Attention, executive function and psychomotor speed**
TMT-A	57.00 (44.00, 89.00)	43.00 (39.00, 59.00)	0.016*^a^
TMT-B	151.00 (120.50, 208.00)	110.00 (95.50, 138.00)	0.006*^a^
**Working memory**
DST forwards	8.24 ± 1.39	10.00 ± 1.35	<0.001*
DST backwards	3.68 ± 1.03	3.72 ± 0.79	0.878
**Semantic memory**
VFT	40.96 ± 8.44	40.24 ± 6.97	0.744
**Episodic memory**
AVLT immediate recall	6.74 ± 1.64	7.10 ± 1.76	0.458
AVLT short term delayed recall	7.04 ± 3.58	7.84 ± 2.73	0.379
AVLT long term delayed recall	5.12 ± 4.16	6.76 ± 2.54	0.099
AVLT long term delayed recognition	9.92 ± 3.72	10.12 ± 2.67	0.828
AVLT total score	28.82 ± 10.98	31.82 ± 8.34	0.282

*p < 0.05. Data are presented as the mean ± SD for normally distributed continuous data and the median (QR) for non-normally distributed data. ^a^The p value was obtained using the Mann-Whitney U test. MMSE, Mini-Mental State Examination; MoCA, Montreal Cognitive Assessment; TMT, Trail Making Test; DST, Digital Span Test; VFT, Verbal Fluency Test; AVLT, Auditory Verbal Learning Test.

### Comparison of Brain Volume Across Groups

The gray matter, white matter and brain parenchyma volumes (the total volumes of gray and white matter) did not differ significantly between the two groups (Table 1).

### ReHo Analysis

**Figure 1:**
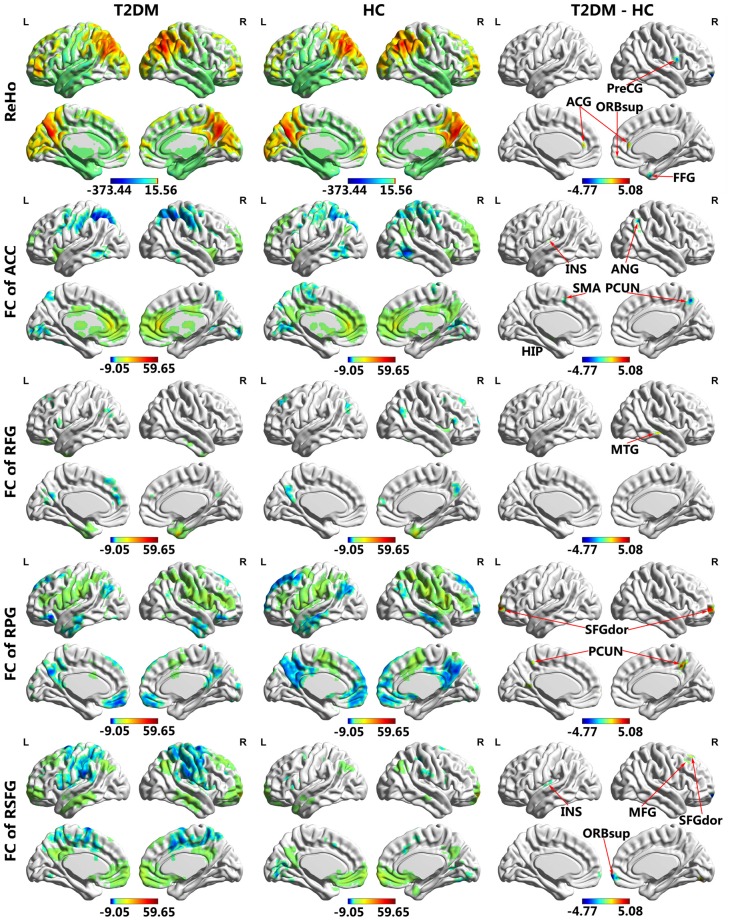
**Regional homogeneity (ReHo) and functional connectivity (FC) maps of intra-group and inter-group comparisons.** Voxel *p* < 0.005 (AlphaSim correction, cluster *p* < 0.01, cluster size >16 voxels). Color scale denotes the *t* value. ACG, anterior cingulate gyrus; FFG, fusiform gyrus; PreCG, precentral gyrus; ORBsupmed, superior frontal gyrus, medial orbital; INS, insula; ANG, angular gyrus; HIP, hippocampus; SMA, supplementary motor area; PCUN, precuneus; MTG, middle temporal gyrus; SFGdor, superior frontal gyrus, dorsolateral; ORBsup, superior frontal gyrus, orbital part; MFG, middle frontal gyrus; R, Right; L, Left.

**Figure 2:**
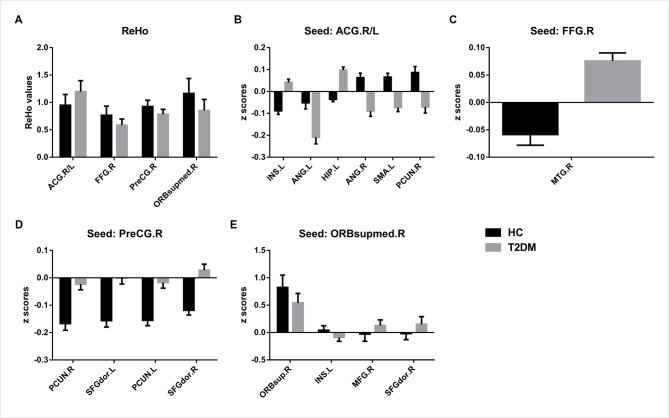
**Brain regions with significant differences in ReHo (A) and FC (B–E) between Type 2 diabetes mellitus (T2DM) patients and healthy controls.** Voxel *p* < 0.005 (AlphaSim correction, cluster *p* < 0.01, cluster size >16 voxels). ACG, anterior cingulate gyrus; FFG, fusiform gyrus; PreCG, precentral gyrus; ORBsupmed, superior frontal gyrus, medial orbital; INS, insula; ANG, angular gyrus; HIP, hippocampus; SMA, supplementary motor area; PCUN, precuneus; MTG, middle temporal gyrus; SFGdor, superior frontal gyrus, dorsolateral; ORBsup, superior frontal gyrus, orbital part; MFG, middle frontal gyrus; R, Right; L, Left.

The T2DM patients exhibited significantly higher ReHo values than the healthy controls in the bilateral anterior cingulate gyrus (ACG) and significantly lower ReHo values in the right fusiform gyrus (FFG), right precentral gyrus (PreCG) and right medial orbital part of the superior frontal gyrus (SFG; Table 3; Figures 1, 2A).

**Table 3:** **Brain regions with different regional homogeneity (ReHo) values between T2DM patients and healthy control groups**.

	Brain regions	BA	Peak MNI			*t* value	Cluster (voxels)	Cluster (mm^3^)
			*X*	*Y*	*Z*
1	R/L Anterior cingulate gyrus	32	0	39	9	3.8304	50	1350
2	R Fusiform gyrus	36	21	3	−42	−4.3742	48	1296
3	R Precentral gyrus	6	54	6	21	−3.9886	34	918
4	R Superior frontal gyrus, medial orbital	11	12	63	−9	−4.6763	28	756

*MNI, Montreal Neurological Institute; BA, Brodmann Area; R, Right; L, left; voxel p < 0.005 (AlphaSim correction, cluster p < 0.01, cluster size >16 voxels)*.

### FC Analysis

The results of the FC group comparisons are presented in Table 4 and Figures 1, 2B–E. The T2DM patients exhibited weaker negative connectivity relative to the healthy controls between the right PreCG and bilateral precuneus. In addition, negative to positive transformations of connectivity were observed in several brain regions, such as the FC between the right FFG and right middle temporal gyrus (MTG). Weaker positive connectivity was observed between the right orbital part and right medial orbit of the SFG. There were also brain regions that experienced positive to negative transformations of connectivity, such as the FC between the ACG and the right precuneus. In addition, the T2DM patients exhibited greater negative connectivity between the ACG and the left angular gyrus (ANG).

**Table 4:** **Brain regions with different functional connectivity (FC) between T2DM patients and healthy control groups**.

Brain regions	BA	Peak MNI			*t* value	Cluster (voxels)	Cluster (mm^3^)
		*X*	*Y*	*Z*			
**FC of R/L Anterior cingulate gyrus**
1 L Insula	48	−33	−15	15	3.6266	17	459
2 L Angular gyrus	7	−36	−60	42	−3.9769	17	459
3 L Hippocampus	20	−30	−15	−9	5.0636	30	810
4 R Angular gyrus	39	42	−60	33	−4.0440	42	1134
5 L Supplementary motor area	6	−12	12	51	−4.1777	16	432
6 R Precuneus	7	3	−66	48	−3.6344	16	432
**FC of R Fusiform gyrus**
1 R Middle temporal gyrus	21	69	−27	−6	4.7849	20	540
**FC of R Precentral gyrus**
1 R Precuneus		12	−48	42	4.3937	37	999
2 L Superior frontal gyrus, dorsolateral	10	−21	66	3	4.4762	35	945
3 L Precuneus	17/23	−15	−54	15	4.3099	43	1161
4 R Superior frontal gyrus, dorsolateral	10	21	57	0	4.4099	35	945
**FC of R Superior frontal gyrus, medial orbital**
1 R Superior frontal gyrus, orbital part	11	15	66	−12	−4.6025	41	1107
2 L Insula	48	−39	−18	18	−4.2647	25	675
3 R Middle frontal gyrus	9	45	24	51	4.1148	20	540
4 R Superior frontal gyrus, dorsolateral	8	27	30	60	4.0556	28	756

*MNI, Montreal Neurological Institute; BA, Brodmann Area; R, Right; L, left; voxel p < 0.005 (AlphaSim correction, cluster p < 0.01, cluster size >16 voxels)*.

### Correlation Analysis

**Figure 3:**
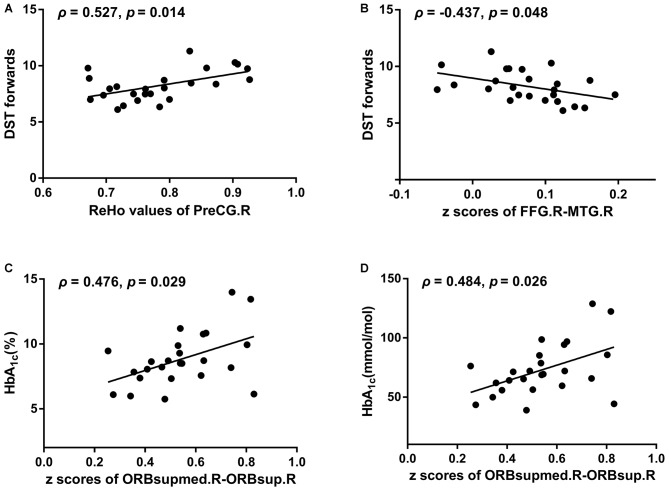
**The relationships of aberrant ReHo/FC with neuropsychological performances/diabetes-related parameters after adjustment for age, sex, education and body mass index (BMI). (A)** ReHo values of PreCG.R vs. DST forwards. **(B)** *z* scores of FFG.R-MTG.R vs. DST forwards. **(C)** *z* scores of ORBsupmed.R-ORBsup.R vs. HbA_1c_ (%). **(D)** *z* scores of ORBsupmed.R-ORBsup.R vs. HbA_1c_ (mmol/mol). DST, Digital Span Test; PreCG, precentral gyrus; FFG, fusiform gyrus; MTG, middle temporal gyrus; ORBsupmed, superior frontal gyrus, medial orbital; ORBsup, superior frontal gyrus, orbital part; R, Right; L, Left.

Better DST forward scores were correlated with higher ReHo values of the right PreCG (*ρ* = 0.527, *p* = 0.014; Figure 3A) and stronger negative FC between the right FFG and the MTG (*ρ* = −0.437, *p* = 0.048; Figure 3B). Higher levels of HbA_1c_ were associated with a stronger positive FC between the right orbital part and the right medial orbit of the SFG (HbA_1c_ [%], *ρ* = 0.476, *p* = 0.029; HbA_1c_ [mmol/mol], *ρ* = 0.484, *p* = 0.026; Figures 3C,D). No significant correlations were observed between the FC of other brain regions and clinical data or cognitive test scores.

## Discussion

It has been proposed that the normal brain development experiences a “local to remote” FC organization pattern with important implications for revealing the neural substrate of cognition (Fair et al., 2009). Previous studies have also reported that local alteration of functional synchronization can diffuse to remote synchronization in diseases such as schizophrenia (Jiang et al., 2015), subjective tinnitus (Chen et al., 2015b) and relapsing-remitting multiple sclerosis (Wu et al., 2016). In the present study, the T2DM patients exhibited abnormal local brain activity synchronization in the bilateral ACG, right FFG, PreCG and medial orbital part of the SFG, as detected by ReHo analysis. Using FC analysis, we further found aberrant brain activity synchronization between the frontal and parietal lobes. These abnormalities suggested that the T2DM patients were subjected to disease attacks, which could lead to brain dysfunction from the local to global scales.

Although the T2DM patients’ general cognition assessed by MMSE and MoCA was not significantly decreased, they indeed suffered from cognitive impairment according to the current rs-fMRI study and neuropsychological test results. Compared to healthy controls, the T2DM patients had decreased ReHo values, and the decreased ReHo values in the right PreCG were associated with lower DST forward scores. These decreases could be harmful to brain function, according to previous studies (Cui et al., 2014; Peng et al., 2016). Furthermore, evidence from a recent functional MRI study showed that the precuneus, which is the central node of the posterior DMN, exhibited decreased connectivity in T2DM patients by local brain independent component analysis (Cui et al., 2015). In the present study, the changed FC between the ACG and the right precuneus might suggest the disassociation of anterior and posterior components of the DMN, which is a major contributor to normal cognitive function (Cui et al., 2015).

More specifically, our findings provide information about the domains of cognitive impairment. The aberrant FC observed between the ACG and right ANG was consistent with previous studies (Zhou et al., 2010; Mormino et al., 2011; Hoogenboom et al., 2014). Decreased FC between the ACG and left hippocampus has been observed in elderly T2DM patients (Zhou et al., 2010) and is also apparent in middle-aged patients. These changed brain areas have been related to episodic memory (Zhou et al., 2010; Mormino et al., 2011), suggesting that episodic memory might be a vulnerable cognitive domain in T2DM patients. Moreover, our findings suggest dysfunction of working memory in T2DM patients. Brain regions, including the supplementary motor area (SMA), parietal areas and hippocampus, which were involved in aberrant FC in the present study, were significantly activated in the spatial n-back task experiment (Huang et al., 2015). Further, with the FC between the right FFG and MTG deviating from the baseline, T2DM patients scored poorer on DST forward tests. This evidence could support the notion that working memory is impaired in T2DM patients. However, the diabetic brain might attempt to maintain cognitive function to overcome the attack by T2DM on memory. The ACG has been reported to have increased neural intensity in the resting-state (Cui et al., 2014) or during a working memory task (He et al., 2015) in T2DM patients. Higher ReHo in the ACG might represent compensatory neural activities for maintaining normal cognition (He et al., 2015). In addition, poor performance on the TMT-A and TMT-B indicated that T2DM patients exhibited executive, attention and psychomotor speed dysfunctions, consistent with previous studies (Chen et al., 2014, 2015a; Cui et al., 2014, 2015). The PreCG is the site of the primary motor cortex, and it is reportedly atrophic in T2DM patients (Chen et al., 2012). Thus, deactivation of the PreCG might account for the slower drawing speed on the TMT-A and TMT-B tests.

Emotion and behavior were also affected by T2DM because certain changed brain regions were involved in these functions, except for cognition. T2DM patients often have treatment-related, recurring periods of low blood glucose levels, which are associated with negative mood states (Anderson et al., 2001). The SFG, SMA, insula and FFG are emotion-associated brain regions vulnerable to blood glucose fluctuations, as concluded from a mood induction experiment (Kohn et al., 2015). Although we excluded subjects with depression, the fMRI results indicated that the remaining patients might be at risk of developing depressive symptoms. Moreover, the insula performs an integrative function in the olfacto-gustatory system (Kurth et al., 2010), and it plays an important role in flavor identification (Rolls, 2006). The insula morphology and energy variability might underlie dysfunction of food intake regulation (Jauch-Chara et al., 2015). Therefore, the altered FC between the ACG and left insula could reflect the ingestion behavior response to blood glucose level changes, which contribute to the typical symptoms of T2DM patients.

According to the results, we attempted to depict the patterns of FC alterations in middle-aged T2DM patients. Except for the stronger negative correlation between the ACG and left ANG, the FC of most abnormal brain regions manifested inverse time course correlations compared to healthy controls, including inverse negative and positive correlations. We inferred that the weakened negative and positive correlations would finally develop into inverse connectivity patterns as the disease progresses. Weaker negative correlations between the right PreCG and bilateral precuneus have also been seen in Alzheimer’s disease (Wang et al., 2007), in agreement with our findings. This finding suggested that these two diseases might share similar mechanisms. Few studies have reported these change patterns in T2DM patients. Either inverse or weakened connectivity patterns tend to be associated with poorer cognitive performance, which might reflect the detrimental effects of T2DM on brain function. Cognitive training can hopefully strengthen the positive or negative correlations of FC as well as reverse the aberrant, reversed positive or negative correlations (Cao et al., 2016). It provides a rewarding method to intervene in cognitive impairment related to T2DM. Moreover, the pattern of FC might be a meaningful imaging marker to detect brain dysfunction and to monitor the results of interventions in T2DM patients.

T2DM is a metabolic disorder and is often accompanied by abnormalities in certain clinical variables. It is difficult to eliminate the influences of these clinical variables on the brain’s functional activity. In the current study, HbA_1c_ levels were positively correlated with the FC between the right orbital part and the medial orbital of the SFG. Our findings were similar to those of a previous study (Hoogenboom et al., 2014). In this study, a positive correlation was found between HbA_1c_ and uncinate fasciculus fractional anisotropy in both the T2DM patients and healthy controls. However, higher uncinate fasciculus fractional anisotropy was associated with increased information processing speed (Hoogenboom et al., 2014), thus appearing to contradict the idea that T2DM patients may benefit from blood glucose control to prevent cognitive decline (Kravitz et al., 2013; Biessels et al., 2014). However, HbA_1c_ only reflects the ambient glucose concentration of the previous 2 to 3 months (O’Keeffe et al., 2016). The duration of FC change may be longer than 2 to 3 months. Therefore, HbA_1c_ might not be the best predictor of the brain’s condition, or the positive relationship might be attributable to a compensatory mechanism in middle-aged patients (He et al., 2015). In summary, this question must be studied further.

Our study has several limitations. First, the relatively small population size might have restricted the ability to investigate the full extent of ReHo and FC. Therefore, the results should be interpreted cautiously. Second, we tried to achieve inter/intra-group homogeneity except for T2DM-related parameters, but inter-group differences existed in terms of HDL cholesterol, serum creatine and FT3 levels, and there was heterogeneity in the drug therapy of the T2DM patients. The influences of these factors on brain function must be studied further. Third, we adopted a traditional method to analyze the FC, which assumed that the connections remained static in the period of MRI data collection. However, dynamic analysis, a method that has recently emerged, revealed the transient connectivity of the brain, which could improve the temporal resolution of fMRI and capture spontaneously jumping changes in thoughts over time (Hutchison et al., 2013).

## Conclusion

This study investigated the spatially local and remote brain activity synchronization in middle-aged T2DM patients. We found that aberrant ReHo and FC were mainly distributed in the insula, MTG and frontal and parietal lobes, which are related to cognitive impairment. It is noteworthy that the patterns of ReHo and FC alterations might serve as imaging biomarkers for the diagnosis and evaluation of the effects of diabetes on the brain. Our findings shed light on the mechanisms underlying T2DM-associated brain dysfunction.

## Author Contributions

DL contributed to performing the experiments, data analysis and writing of the manuscript. SD contributed to performing the experiments and revising the manuscript. JZ designed the experiment and revised the manuscript. CZ contributed to the data collection. ML contributed to the data analysis and manuscript revision. XY contributed to the manuscript revision. PW and JW are the guarantors of this work and, as such, had full access to all of the data in the study, and they accept responsibility for the integrity of the data and the accuracy of the data analysis.

## Funding

The study was supported by the National Natural Science Foundation of China (81471647) and the Innovation Fund for Younger Investigators of Southwest Hospital of the Third Military Medical University (SWH2013QN09).

## Conflict of Interest Statement

The authors declare that the research was conducted in the absence of any commercial or financial relationships that could be construed as a potential conflict of interest.

## Footnotes

^1^http://www.idf.org/publications/annual-report

^2^http://www.fil.ion.ucl.ac.uk/spm

^3^http://www.mathworks.com

^4^http://www.restfmri.net/forum/DPARSF

^5^http://www.restfmri.net
